# Herpes Simplex Virus 1 in Trigeminal Ganglia of Trafficked Neotropical Primates, Peru, 2024

**DOI:** 10.3201/eid3205.251408

**Published:** 2026-05

**Authors:** Fernando Vilchez-Delgado, Lin Zhou, Shannon O’Connor, Renato Colan, Leticia Escobar-Mendoza, A. Patricia Mendoza, Bruno M. Ghersi, Roy Andrade, Michael Talledo-Albújar, Marieke H. Rosenbaum

**Affiliations:** Cummings School of Veterinary Medicine, North Grafton, Massachusetts, USA (F. Vilchez-Delgado, L. Zhou, S. O’Connor, B.M. Ghersi, M.H. Rosenbaum); Universidad Peruana Cayetano Heredia, San Martin de Porres, Lima, Peru (F. Vilchez-Delgado, L. Escobar-Mendoza, R. Andrade, M. Talledo-Albujar); National Forest and Wildlife Service, Lima (R. Colan); Washington University–St. Louis, St. Louis, Missouri, USA (A.P. Mendoza)

**Keywords:** herpes simplex virus 1, viruses, zoonoses, Neotropical primates, alphaherpesvirus, monkeys, wildlife trafficking, latency, Peru

## Abstract

We detected herpes simplex virus 1 in the trigeminal ganglia of trafficked Neotropical primates (1 *Aotus* *azarai*; 3 *Sapajus* *macrocephalus*) in Peru. Tests also revealed *Saimiriine alphaherpesvirus* 1 in the trigeminal ganglia of 2 *Saimiri* sp. monkeys. Our findings suggest latency and raise concerns about diagnostic standards, viral reactivation, and spillover risks.

Illegal trafficking operations have lead to the extraction of thousands of Neotropical primates (NP) from the Peruvian Amazon, exposing them to humans and other domestic and wild species, creating opportunities for bidirectional zoonotic disease transmission and spillover (*1*). Among humans, herpes simplex virus 1 (HSV-1) infects around 67% of adults globally (*2*) and establishes lifelong latency in the trigeminal ganglia (TG) (*3*). HSV-1 infections in NP can cause mild or severe disease, sometimes leading to ulcerative lesions and neurologic impairment (*4*). Questions remain, however, regarding the ability of HSV-1 to establish latency in NP after natural infection.

During latency in humans, HSV-1 lytic gene expression is suppressed, but the latency-associated transcripts and associated microRNAs remain transcriptionally active (*5*). As a result, production of infectious viral particles ceases (*6*), and diagnostic approaches based on PCR testing of peripheral tissues, blood, or oral swab samples fail to detect latently infected persons. Trafficked NP with undetected HSV-1 infections might be placed into rehabilitation centers and possibly released into wild populations, posing a threat to primate health and conservation (*7*,*8*).

To assess whether HSV-1 establishes latency in NP, we investigated the presence of viral DNA in the TG (and its absence in other tissues) in 37 trafficked NP carcasses representing 7 species (Appendix Table 1) in Peru. On gross examination, we observed no lesions suggestive of active HSV-1 infection, such as oral mucosal ulcers, and we classified all animals as asymptomatic on the basis of macroscopic findings alone.

We aseptically collected oral swab samples as well as TG, heart, liver, spleen, kidney, and salivary gland samples and preserved all samples in RNAlater (Thermo Fisher Scientific, https://www.thermofisher.com). When fresh carcasses were available, we preserved TG in 10% formalin for histologic confirmation (Appendix Figures 1, 2). In extracting DNA, we used a nested panherpesvirus PCR, targeting a 215–315 bp region of the DNA polymerase gene, as previously described (*9*) (Appendix). TG samples were positive for herpesvirus DNA (Appendix Table 2) in 13 (35%) NP. Sequencing results revealed *Alphaherpesvirinae* DNA from 6 of the samples and *Gammaherpesvirinae* DNA from the other 7 samples.

We identified HSV-1 in the TG of 4 (11%) NP sampled: 1 *Aotus* *azarai* monkey and 3 *Sapajus* *macrocephalus* monkey. We noted no HSV-1 in any of the 35 oral swab samples we analyzed, including those from TG-positive primates. In 2 of the 4 NP with HSV-1–positive TG, testing also revealed HSV-1 in the spleen, kidney, or both (Table). We observed *Saimiriine herpesvirus* 1, another alphaherpesvirus species, in the TG of 2 *Saimiri* monkeys.

**Table:** Herpesvirus detection in various organs and sample sites from trafficked Neotropical primates with *Alphaherpesvirus*-positive trigeminal ganglia, Peru, 2024

Animal ID	Species	Sample type						
		Trigeminal ganglia	Oral swab	Heart	Liver	Spleen	Kidney	Salivary glands
NE-004-24	*Sapajus*	HSV-1	SapLCV1	SapLCV1	NR	NR	NR	SapCMV1
NE-015-24	*Sapajus*	HSV-1	NR	NR	NR	NR	NR	NR
NE-020-24	*Aotus*	HSV-1	NR	NR	NR	HSV-1	HSV-1	NR
NE-033-24	*Sapajus*	HSV-1	NR	NR	NR	HSV-1	NR	NR
NE-011-24	*Saimiri*	SaHV-1	SsciLCV2	SsciLCV2	NR	NR	NR	SsciCMV1
NE-021-24	*Saimiri*	SaHV-1	SbolCMV1	SsciLCV2	NR	SsciLCV2	NR	SbolCMV1

*ID, identification; HSV-1, human simplexvirus 1; NR, negative results; SaHV-1, *Saimiriine* herpesvirus 1; SapCMV1, *Sapajus apella* cytomegalovirus 1; SapLCV1, *Sapajus apella* lymphocryptovirus 1; SbolCMV1, *Saimiri boliviensis* cytomegalovirus 1; SsciCMV1, *Saimiri sciureus* cytomegalovirus 1; SsciLCV2, *Saimiri sciureus* lymphocryptovirus 2.

Phylogenetic analysis of the full HSV-1 DNA polymerase gene (≈4 kb) revealed that 2 sequences (from primates NE-004-24 and NE-033-24) share a recent common ancestor. The remaining sequences are distributed across different branches of the phylogenetic tree, suggesting the infections originated from genetically distinct introductions (spillovers) rather than from a localized outbreak (Figure).

**Figure:**
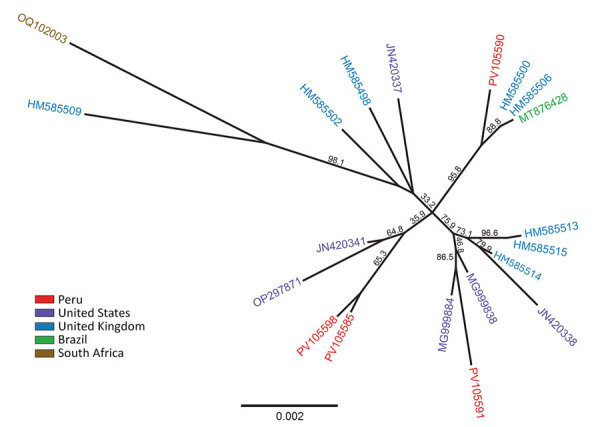
Maximum-likelihood phylogeny of herpes simplex virus 1 (HSV-1) created for study of HSV-1 in trigeminal ganglia of trafficked neotropical primates, Peru, 2024. Tree constructed from ≈4 kb of the UL30 DNA polymerase gene, applying the general time reversible substitution model with 1,000 bootstrap replicates. Red text indicates full-length HSV-1 DNA polymerase gene sequences detected in trigeminal ganglia of 4 primates from Peru. For comparison, we selected 16 additional reference sequences from GenBank (accession numbers provided) to represent diverse geographic regions worldwide. Phylogenetic analysis suggests a genetically diverse origin of HSV-1 infections in the primates evaluated, likely derived from genetically distinct introductions (spillovers). Sequences deposited in GenBank (accession nos. PV105585 [primate NE-004-24], PV105590 [primate NE-015-24], PV105591 [primate NE-020-24], and PV105598 [primate NE-033-24]).

Multiorgan analysis revealed co-infections with 3 distinct herpesviruses, representing all herpesvirus subfamilies (*Alphaherpesvirinae*, *Betaherpesvirinae*, and *Gammaherpesvirinae*), in 3 TG-positive NP (Table). We noted cytomegaloviruses in the salivary glands of 3 of the TG-positive NP, as well as lymphocryptoviruses in the heart tissue, spleen, or both of those same 3 primates. We did not assess the herpesvirus status of organs from TG-negative NP.

Our findings suggest that, as in the case of human infections, HSV-1 may naturally establish latency in the TG of some NP. The detection of HSV-1 DNA in the spleen and kidney does not rule out latency, because latency also has been documented in nonneuronal cells, including neutrophils and B and T lymphocytes (*10*). Confirming true latency in NP would require herpesvirus reactivation studies and RNA sequencing from positive TG.

None of the NP with HSV-1–positive TG showed macroscopic lesions compatible with HSV-1 disease, and we detected no viral DNA in oral swabs. Those findings highlight a critical diagnostic challenge in detecting latent HSV-1 infections in live NP. Oral swab samples can be collected from living primates with minimal distress, but TG can only be obtained postmortem, precluding their use in health evaluations before releasing animals into the wild. Whether latently infected NP can undergo viral reactivation under natural stress conditions and transmit HSV-1 to humans or naive NP remains unknown.

In conclusion, we detected HSV-1 and *Saimiriine herpesvirus* 1 in the TG of NP, consistent with latency. Our findings underscore the relevance of TG as a target tissue for future research and broaden our understanding of the diversity and latency of alphaherpesviruses in NP. Our study also highlights the need for less invasive methods, such as specific antibody profiles or T-cell–specific biomarkers of latency, to identify latent herpesvirus infections in live NP. Identifying such infections can help mitigate potential spillover to other primates, including humans.

Appendix Additional information for herpes simplex virus 1 in trigeminal ganglia of trafficked neotropical primates, Peru, 2024.
