# Supplementary material for: Herpes Simplex Virus 1 in Trigeminal Ganglia of Trafficked Neotropical Primates, Peru, 2024
Source: Emerg Infect Dis. 2026 May;32(5):811–4. doi: 10.3201/eid3205.251408 (PMC13174968; doi:10.3201/eid3205.251408)
Supplement: Appendix — Additional information for herpes simplex virus 1 in trigeminal ganglia of trafficked neotropical primates, Peru, 2024. [file 25-1408-Techapp-s1.pdf]

Article DOI: http://doi.org/10.3201/eid3205.251408

EID cannot ensure accessibility for supplementary materials supplied by authors. Readers who have difficulty accessing supplementary content should contact the authors for assistance.

Herpes Simplex Virus 1 in Trigeminal Ganglia of Trafficked Neotropical Primates, Peru, 2024

Appendix

Materials and methods

Necropsies, IACUC, and research permits

We performed necropsies on 37 NP confiscated from wildlife trafficking and euthanized by Peruvian authorities due to reasons unrelated to this project and in accordance to their internal protocols for the placement of confiscated wildlife. Our study was conducted under the research permit AUT-IFS-2020–056 granted by the Peruvian Wildlife and Forestry Service (SERFOR). Sample collection and necropsy protocols were approved by Tufts University's (G2023–40) and Universidad Peruana Cayetano Heredia's IACUC (SIDISI 103076).

Collection of trigeminal ganglia and other samples

Out of the 37 carcasses, 35 oral swabs were available, including 27 that were collected immediately before euthanasia and 8 collected postmortem following freezing. Necropsies were performed to aseptically collect the following specimens in RNAlater: oral swabs, TG, heart, liver, spleen, kidney, and salivary glands. The collection of TG was guided by previously described methods (*1*). In short, after removal of the top of the skull and brain, the TG were found immediately ventral to the dura mater before it splits into 3 branches and exits the skull (Appendix Figure 1, panels A–C). When fresh carcasses were available, the TG were removed and preserved in 10% formalin for confirmation via histology using hematoxylin and eosin standard staining (Appendix Figure 1, panel D). An illustrative protocol for collecting TG is depicted in Appendix Figure 2.

DNA extraction and quality control

DNA was extracted from samples using a commercial kit (Roche High Pure PCR Template Preparation Kit, Roche, Germany). Tissues were incubated in lysis buffer and proteinase K overnight, then disrupted by shaking with a Disruptor Genie machine (Scientific industries, Inc, USA). PCR targeting the primate mitochondrial housekeeping gene Cytochrome B was conducted to confirm successful DNA extraction (*2*).

Herpesvirus PCR screening

To detect herpesvirus DNA among samples, we used a nested pan-herpesvirus PCR assay targeting a 215–315 bp region of the DNA polymerase gene, as previously described (*3*). This protocol is designed to detect members of the *Alpha*-, *Beta*-, and *Gammaherpesvirinae* subfamilies. The first-round amplification used primers DFA (TGGTNNTNGAYTTYGCNAGYYTNTAYCC), KG1 (GTCTTGCTCACCAGNTCNACNCCYTT), and ILK (TCCTGGACAAGCAGCARNYSGCNMTNAA). The second-round amplification used primers TGV (TGTAACTCGGTGTAYGGNTTYACNGGNGT) and IYG (CACAGAGTCCGTRTCNCCRTADAT).

PCR amplification was performed using Platinum Taq DNA Polymerase (Invitrogen, USA), following the manufacturer's instructions with minor modifications, including a final primer concentration of 0.2 μM and the addition of 5% dimethyl sulfoxide (DMSO). Template DNA input volume was 3 μL. Each PCR reaction was carried out in a final volume of 25 μL. Thermal cycling conditions for both amplification rounds consisted of an initial denaturation at 94°C for 120 seconds, followed by 45 cycles of denaturation at 94°C for 30 seconds, annealing at 52°C for 60 seconds, and extension at 72°C for 60 seconds, with a final extension step at 72°C for 10 minutes.

PCR products were resolved on 2% agarose gels prepared in 0.5× TBE buffer and stained with either ethidium bromide (0.5 μg/mL) or GelRed (1 μL per 10 mL of gel solution). Electrophoresis was performed in 0.5× TBE buffer at 100 V for ≈60 minutes. Amplicon size was estimated by comparison with a 100 bp DNA ladder, and products within the expected 215–315 bp size range were considered presumptively positive.

All samples were tested in duplicate. A sample was considered positive only if a band of the expected size was observed in at least one replicate and subsequently confirmed by Sanger sequencing. For individuals testing positive for alphaherpesviruses in the TG, additional tissues including heart, liver, spleen, kidneys, and salivary glands were subsequently screened for herpesviruses using the same nested PCR protocol to assess tissue distribution of viral DNA.

Sequencing and viral species identification

Positive PCR amplicons were purified and sequenced using Sanger Sequencing through a commercial service (Azenta, MA, USA) to confirm the species of the detected herpesviruses. Sequencing was performed using the forward and reverse primers TGV and IYG, respectively (*3*). Raw Sanger sequencing chromatograms were visually inspected in Chromas v 2.6.6 (Technelysium Pty Ltd). Primer sequences were trimmed manually, and low-quality bases at the 5′ and 3′ ends were removed before consensus generation. Forward and reverse reads were assembled and manually curated using Mega X version 10.2.6 (*4*). Ambiguous base calls were resolved by reviewing chromatogram signal quality. Only high-quality consensus sequences were retained for downstream analyses. Consensus sequences were deposited in NCBI GenBank under accession numbers PV105585 to PV105607.

To determine viral species identity, consensus sequences were compared against reference sequences in the NCBI database using the BLASTn algorithm. Viral species assignment was based on highest sequence identity scores.

Long PCR amplification of HSV-1 UL30 gene sequences

The UL30 (DNA polymerase) gene sequences of herpes simplex virus 1 (HSV-1) were amplified from positive trigeminal ganglia that tested positive for HSV-1 by nested pan-herpesvirus PCR. A nested PCR approach was used to amplify an ≈4 kb fragment encompassing the entire UL30 coding region, as previously described (*5*). The first round of amplification employed primers POL1-F1 (CGGCTACGTCACKCTCCTGT) and POL1-R1 (CGGAGATTCCGACCGTGT). The second round used primers POL1-F2 (GCCGACGCGAATAAACC) and POL1-R2 (GGCCGCAGACATTTATTGTAA).

PCR reactions were performed using the Platinum SuperFi II High-Fidelity DNA Polymerase (Invitrogen, USA), following the manufacturer's recommendations. Each reaction contained a final primer concentration of 0.5 μM. Thermal cycling conditions for both rounds

consisted of an initial denaturation at 98°C for 30 seconds; 40 cycles of denaturation at 98°C for 15 seconds, annealing at 60°C for 15 seconds, and extension at 72°C for 90 seconds; followed by a final extension at 72°C for 10 minutes. Amplification products were visualized on 2% agarose gels as described in the 'Herpesvirus PCR Screening section'.

Subsequently, the second amplification product was used to re-amplify overlapping sections to fully cover the DNA Polymerase region. The primers used were POL1-F2 + POL1-A (GCTTGAGGTGACCGTCGT), POL1-B (CGTACTATAGCGAATGCGATGA) + POL1-C (GAAGTGGTCCGCGGAGAT), POL1-D_edit (GGTYGACAGGCACCTACAAT) + POL1-G (CACGGCGTTGAGCTTGTAG), POL1-F (AGGACGAGCTGGCCTTTC) + POL1-G, POL1-H (GTGTGGGACATAGGCCAGAG) + POL1-I (GTCCTCCCCCTCCTCCTC), POL1-J (CCGACCAGAAGGGCTTTATT) + POL1-K (GCTGCTTGTCCAGGAGCAC), POL1-L (CCATGCGAAAGCAGATCC) + POL1-M (TCTTACCCCCGTAGATGACG), POL1-N (ATCAAACTCGAGTGCGAAAA) + POL1-O (GCGACCGTCTCCTCTACCTC), and POL1-P (CAGGTCCCGTCCATCAAG) + POL1-R2 (GGCCGCAGACATTTATTGTAA).

PCR conditions for amplification of overlapping fragments consisted of an initial denaturation at 98°C for 30 seconds; 40 cycles of 98°C for 15 seconds, 60°C for 15 seconds, and extension at 72°C for 30 seconds; followed by a final extension at 72°C for 10 minutes. All amplicons were confirmed by agarose gel electrophoresis before sequencing.

Each overlapping fragment was purified and subjected to bidirectional Sanger sequencing using its corresponding forward and reverse primers. Chromatogram processing, quality trimming, and consensus generation were performed as described in the 'Sequencing and viral species identification' section. The complete ~4.0 kb UL30 sequence for each sample was reconstructed by manually inspecting and assembling overlapping fragments in MEGA X version 10.2.6. Four full DNA Polymerase gene sequences were generated in this study and were deposited in GenBank under the accession numbers PV105585 (animal NE-004–24), PV105590 (animal NE-015–24), PV105591 (animal NE-020–24), and PV105598 (animal NE-033–24).

Alignment and phylogenetic analysis

Twenty UL30 (DNA polymerase) gene sequences of herpes simplex virus 1 (HSV-1), each ≈4.0 kb in length, were included in the phylogenetic analysis including the four sequences were generated in this study (PV105585, PV105590, PV105591, and PV105598), and sixteen

reference sequences were retrieved from GenBank (HM585498, HM585500, HM585502, HM585506, HM585509, HM585513, HM585514, HM585515, JN420337, JN420338, JN420341, MG999838, MG999884, MT876428, OP297871, and OQ102003). Reference sequences were selected to represent diverse geographic regions worldwide for which complete HSV-1 UL30 gene sequences were publicly available.

Multiple sequence alignment was performed using MAFFT v7.490 (*6*). Alignment was conducted using the 200 PAM scoring matrix (K = 2), with a gap opening penalty of 1.53 and an offset value of 0.123. Default iterative refinement settings were applied. The resulting alignment was manually inspected to confirm correct codon alignment. The final curated alignment had a length of 4011 bp. The best-fit nucleotide substitution model was determined using jModelTest (version 2.1.10) under the Akaike Information Criterion (AIC) (*7*). The General Time Reversible model (GTR) was identified as the optimal model.

Maximum likelihood (ML) phylogenetic reconstruction was performed using PHYML v3.3.20180621 under the GTR substitution model with four gamma-distributed rate categories and empirical base frequencies (*8*). Branch support was assessed using 1,000 bootstrap replicates. Because no outgroup sequences were included, the resulting phylogeny was midpoint rooted. The final tree was visualized and annotated in Geneious Prime 2024.0.4.

References

1. Howroyd PC. Dissection of the trigeminal ganglion of nonrodent species used in toxicology studies. Toxicol Pathol. 2020;48:30–6. PubMed https://doi.org/10.1177/0192623319854338

2. Smith MF, Patton JL. Phylogenetic relationships and the radiation of sigmodontine rodents in South America: Evidence from cytochrome b. J Mamm Evol. 1999;6:89–128. https://doi.org/10.1023/A:1020668004578

3. VanDevanter DR, Warrener P, Bennett L, Schultz ER, Coulter S, Garber RL, et al. Detection and analysis of diverse herpesviral species by consensus primer PCR. J Clin Microbiol. 1996;34:1666–71. PubMed https://doi.org/10.1128/jcm.34.7.1666-1671.1996

4. Kumar S, Stecher G, Li M, Knyaz C, Tamura K. MEGA X: Molecular evolutionary genetics analysis across computing platforms. Mol Biol Evol. 2018;35:1547–9. PubMed https://doi.org/10.1093/molbev/msy096

5. Burrel S, Deback C, Agut H, Boutolleau D. Genotypic characterization of UL23 thymidine kinase and UL30 DNA polymerase of clinical isolates of herpes simplex virus: natural polymorphism and mutations associated with resistance to antivirals. Antimicrob Agents Chemother. 2010;54:4833–42. PubMed https://doi.org/10.1128/AAC.00669-10

6. Katoh K, Standley DM. MAFFT multiple sequence alignment software version 7: improvements in performance and usability. Mol Biol Evol. 2013;30:772–80. PubMed https://doi.org/10.1093/molbev/mst010

7. Posada D. jModelTest: phylogenetic model averaging. Mol Biol Evol. 2008;25:1253–6. PubMed https://doi.org/10.1093/molbev/msn083

8. Guindon S, Dufayard JF, Lefort V, Anisimova M, Hordijk W, Gascuel O. New algorithms and methods to estimate maximum-likelihood phylogenies: assessing the performance of PhyML 3.0. Syst Biol. 2010;59:307–21. PubMed https://doi.org/10.1093/sysbio/syq010

Appendix Table 1. Demographic data and herpesvirus status of primate carcasses evaluated in Lima, Peru*

Characteristic	Total, n (%)	Positive to herpesvirus in TG, n (%)	Positive to αHV in TG, n (%)
Genus			
Aotus	2 (5.4)	1 (50)	1 (50)
Ateles	4 (10.8)	0 (0)	0 (0)
Cebus	3 (8.1)	0 (0)	0 (0)
Lagothrix	3 (8.1)	1 (33.3)	0 (0)
Leontocebus	6 (16.2)	3 (50)	0 (0)
Saimiri	8 (21.6)	5 (62.5)	2 (25)
Sapajus	11 (29.7)	3 (27.3)	3 (27.3)
Sex			
M	17 (45.9)	3 (17.6)	1 (5.9)
F	20 (54.1)	10 (50)	5 (25)
Age group			
Infant	2 (5.4)	0 (0)	0 (0)
Juvenile	16 (43.2)	7 (43.8)	4 (25)
Adult	19 (51.4)	6 (31.6)	2 (10.5)

*The positivity percentage reflects the proportion of individuals in each category that tested positive for herpesviruses or αHV in the TG. Abbreviations: TG, trigeminal ganglia, αHV, alphaherpesvirus.

Appendix Table 2. Summary of herpesviruses detected in oral swabs and trigeminal ganglia from Neotropical primates*

Animal ID	Demographic data	Viral species in trigeminal ganglia (ID%)	Viral species in oral swab (ID%)
NE-003–24	*Saimiri* sp., female, adult	Saimiriine herpesvirus 2 (96.15)	Not tested
**NE-004–24**	***Sapajus* sp., female, adult**	**Human alphaherpesvirus 1 (100)**	**Cebus apella Lymphocryptovirus 1 (98.75%)**
NE-007–24	*Lagothrix* sp., female, juvenile	Lagothrix lagotricha Lymphocryptovirus 1 (98.83)	Ateles paniscus cytomegalovirus (88.62%)
**NE-011–24**	***Saimiri* sp., female, juvenile**	**Saimiriine herpesvirus 1 (100)**	**Saimiri sciureus lymphocryptovirus 2 (86.67)**
NE-013–24	*Saimiri* sp., female, juvenile	Saimiri sciureus lymphocryptovirus 2 (94.55)	Saimiri sciureus lymphocryptovirus 2 (94.55)
**NE-015–24**	***Sapajus* sp., female, adult**	**Human alphaherpesvirus 1 (100)**	Negative result
**NE-020–24**	***Aotus* sp., male, juvenile**	**Human alphaherpesvirus 1 (100)**	Negative result
**NE-021–24**	***Saimiri* sp., female, juvenile**	**Saimiriine herpesvirus 1 (100)**	**Saimiri boliviensis boliviensis cytomegalovirus (98.84)**
NE-025–24	*Leontocebus* sp., male, juvenile	Leontopithecus rosalia lymphocryptovirus 1 (79.19)	Negative result
NE-027–24	*Leontocebus* sp., female, adult	Saguinus oedipus Lymphocryptovirus 1 (80.11)	Saguinus oedipus Lymphocryptovirus 1 (80.11)
NE-028–24	*Leontocebus* sp., male, adult	Leontopithecus rosalia lymphocryptovirus 1 (78.61)	Leontopithecus rosalia lymphocryptovirus 1 (78.03)
NE-029–24	*Saimiri* sp., female, adult	Saimiriine Herpesvirus 3 (97.66)	Saimiriine Herpesvirus 3 (97.66)
**NE-033–24**	***Sapajus* sp., female, juvenile**	**Human alphaherpesvirus 1 (100)**	Negative result

*Individuals in which alphaherpesviruses were detected are highlighted in bold.
ID% = Identity percentage based on sequence alignment results obtained through NCBI BLAST analysis.

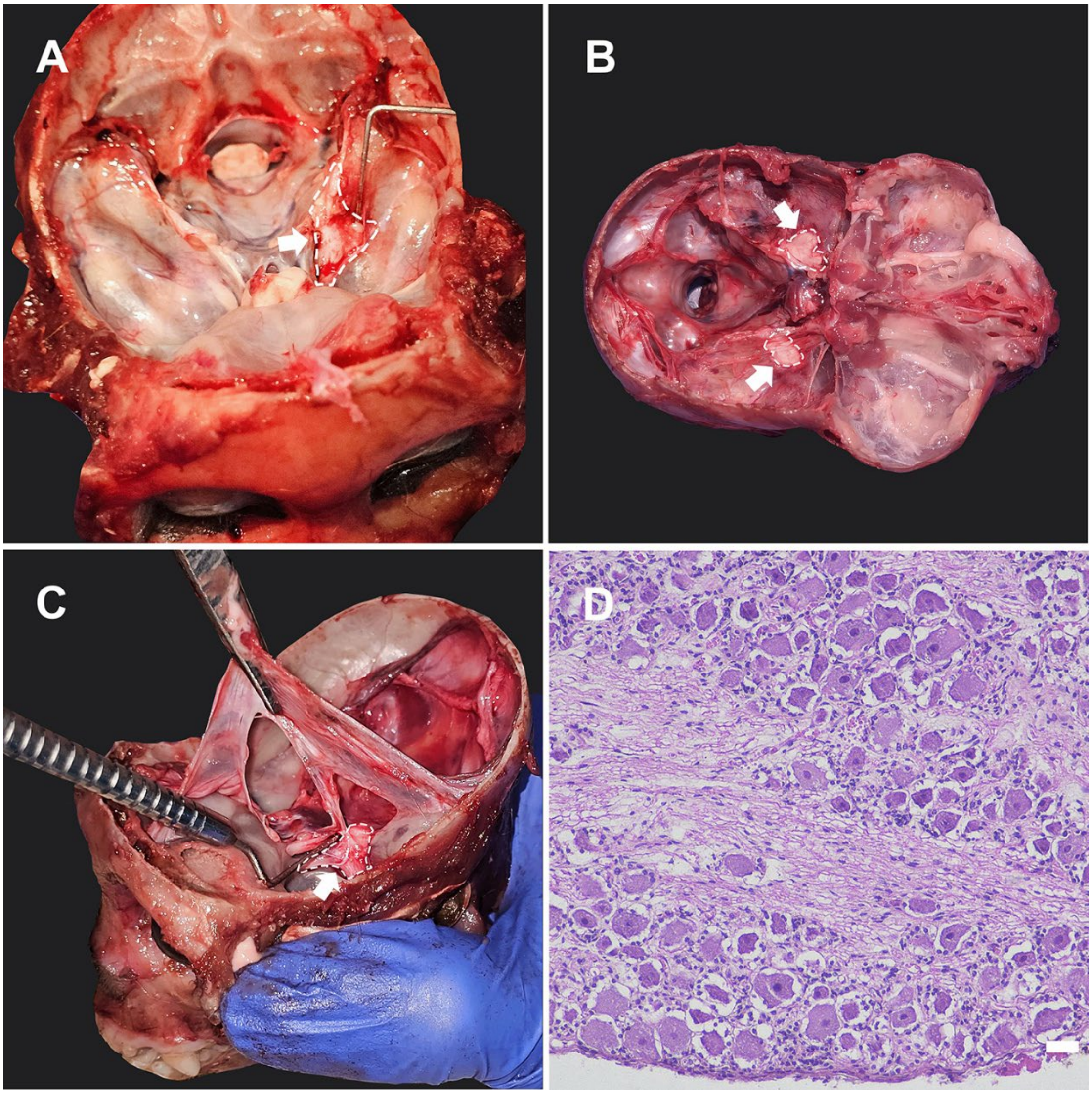

Appendix Figure 1. Localization and histologic confirmation of the trigeminal ganglia in Neotropical primate species. Panels A, B, and C illustrate the anatomic position of the trigeminal ganglia within the skulls of Neotropical primates. The trigeminal ganglion is characterized by a white-yellowish coloration and is located beneath the dura mater in a depression of the temporal bone known as the trigeminal cave, which is medially positioned within the middle cranial fossa. (A) *Sapajus macrocephalus* skull. The dura mater of a tufted capuchin monkey was carefully dissected to expose the left trigeminal ganglion, which is outlined with a dashed line and indicated by an arrow. The right trigeminal ganglion remains concealed beneath the dura mater. (B) Aotus sp. skull. In owl monkeys, the trigeminal ganglion is readily visible before dura mater removal. The structure is highlighted with a dashed line and an arrow. (C) *Cebus* sp. skull. Dissection of the dura mater reveals the left trigeminal ganglion, with its characteristic trifurcation into the ophthalmic, maxillary, and mandibular branches. The ganglion is outlined with a dashed line and indicated by an arrow. (D) Histological confirmation of the trigeminal ganglion in H&E staining, 10x. Scale bar represents 100 μm.

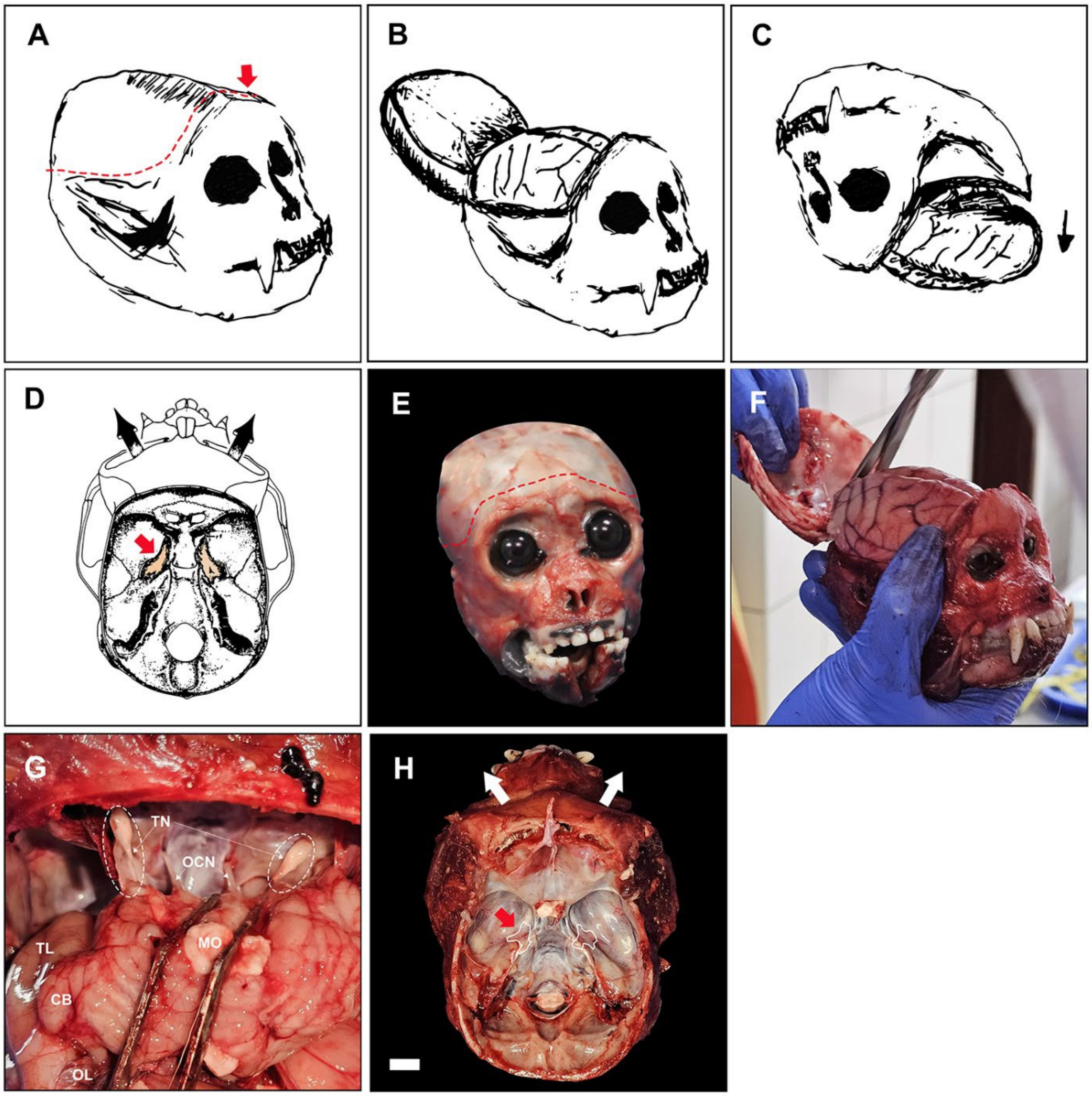

Appendix Figure 2. Collection of Trigeminal Ganglia from Neotropical Primate Carcasses. Panels A–D illustrate a schematic representation of the anatomic position and procedure for trigeminal ganglia (TG) collection, while panels E–H depict the same procedure performed on primate carcasses. (A and E) The skull is dissected, and the skin removed to allow for proper skull opening through the frontal bone, following the dashed line. Panel E shows the skull of a woolly monkey (*Lagothrix* sp.). (B and F) The roof of the skull is removed to expose the brain. Panels F–H show the procedure performed on a tufted capuchin monkey (*Sapajus* sp.). (C and G) The skull is inverted to allow the brain to slide out by gravity. Nerve and vessel connections to the brain are carefully severed. Panel G depicts an inverted caudal-cranial view of the skull, highlighting key anatomic landmarks for identifying and removing the TG. Trigeminal nerves are marked with dashed circles. (D and H) A dorsal-ventral view of the dissected skull

with the roof removed. The orientation of the eyes is indicated by the two upper arrows. The TG are positioned lateral to the cavernous sinus, within a depression of the temporal bone beneath the dura mater. Panel D highlights the exact location of the TG, shown as cream-colored structures marked by an arrow, figure adapted from Hershkovitz 1977. In Panel H, the TG remain concealed beneath the dura mater, a whitish meningeal membrane covering the skull interior. Silhouettes of both TG outlined in white. Scale bar = 1 cm. Abbreviations: TN = Trigeminal nerve, OCN = Oculomotor nerve, MO = Medulla oblongata, TL = Temporal lobe, CB = Cerebellum, OL = Occipital lobe.
